# Impact of Short-Term Gala Apple Intake on the Human Faecal Metabolome Assessed by ^1^H NMR Spectroscopy

**DOI:** 10.3390/nu18091312

**Published:** 2026-04-22

**Authors:** Chandrama Roy Chowdhury, Anna Mascellani Bergo, Eliška Jeníčková, Šárka Knížková, Jaroslav Havlík

**Affiliations:** Department of Food Science, Czech University of Life Sciences Prague, 165 00 Prague, Czech Republic; roy_chowdhury@af.czu.cz (C.R.C.); mascellani@af.czu.cz (A.M.B.); jenickova@af.czu.cz (E.J.);

**Keywords:** fruit, faecal metabolomics, pectin fermentation, colonic digestion, dietary intervention

## Abstract

**Background/Objectives**: Apples are nutritionally valued for their dietary fibre and polyphenols, which influence gut microbial metabolism. However, the metabolic consequences of short-term apple consumption on the human gut environment have not yet been thoroughly investigated. This study aimed to investigate the impact of short-term supplementation of the habitual diet with Gala apples on the human faecal metabolomic profile and to identify metabolite changes reflecting microbial fermentation processes. **Methods**: A pilot dietary intervention was conducted in 15 healthy adults (6 females, 9 males; age 18−30 years). Participants consumed three Gala apples per day as part of their habitual diet for three consecutive days, one with each main meal. Faecal samples were collected before and after the intervention. Metabolic profiling was performed using ^1^H NMR spectroscopy. **Results**: Multivariate analysis showed no clear clustering between pre- and post-intervention samples, suggesting that inter-individual variability exceeded the overall intervention effect. Univariate analysis showed a nominal increase in faecal methanol levels post-intervention (β = +0.72, FC ≈ 2.05, *p* = 0.012); however, this change did not remain statistically significant after correction for multiple testing (q = 0.27). Several other metabolites showed nominal decreases following the intervention, including amino acids, branched-chain fatty acids, short-chain fatty acids, and aromatic microbial metabolites. **Conclusions**: Short-term Gala apple consumption was associated with nominal, metabolite-specific changes in the faecal metabolome without clear global shifts. These findings may show immediate metabolic responses to increased intake of apple-derived substrates; however, given the exploratory design, small sample size, and high inter-individual variability, causal relationships cannot be established. Further studies in larger, controlled cohorts incorporating complementary approaches are required for confirmation.

## 1. Introduction

Diet plays a crucial role in shaping the gut health by influencing the gut microbiota and their metabolic outputs [1]. Fruits are a key source of dietary fibre and bioactive compounds that modulate the microbial activity in the gut environment, contributing to improved digestion and reduced risk of metabolic syndromes [2,3,4,5,6].

Apples (the fruit of *Malus* × *domestica* Borkh) are one of the most commonly consumed fruits because of their diverse cultivars, availability, and unique nutritional qualities [7]. Epidemiological and clinical studies consistently demonstrate that apple consumption is associated with beneficial health outcomes, such as improved lipid profiles, reduced risk of cardiovascular disorders, and diminished markers of systemic inflammation [8,9,10,11,12]. Randomised studies have demonstrated that regular apple intake lowers serum cholesterol, decreasing circulating inflammatory mediators, such as interleukin-6 and *C*-reactive protein, and increasing plasma antioxidant capacity [11,12,13,14]. These benefits could be attributed to the high content of polyphenols and dietary fibres in apples, which show synergistic effects on microbial metabolism and host physiology [2].

A medium-sized apple provides approximately 2–3% dietary fibre, which satisfies 10–15% of the recommended daily intake of dietary fibre [15,16,17]. Out of this dietary fibre, pectin is the major soluble component of the pulp and insoluble fractions such as cellulose, hemicellulose, and protopectin, predominantly in the skin of the apples [18,19,20]. Pectin as a fermentable substrate is important for colonic bacteria, contributing to short-chain fatty acid (SCFA) production, metal chelation, improved bowel motility and gastrointestinal tract wellbeing [3,21,22]. Moreover, polyphenols and fibre synergistically promote saccharolytic fermentation and enhance SCFA production, while reducing proteolytic metabolites such as indoles, *p*-cresol, and to a lesser extent, biogenic amines, shifting colonic fermentation from proteolysis towards saccharolysis [23,24]. Additionally, apples are also major sources of flavonoids, a subgroup of polyphenols, such as quercetin, epicatechin, and procyanidin, which exhibit only low-to-moderate absorption in the small intestine, mostly after deglycosylation by intestinal enzymes [19,25]. The undigested polyphenols are transformed by the gut microbes into low-molecular-weight phenolic acids that maybe more bioactive than their precursors and have greater bioavailability [25]. This modulation influences microbial pathways involved in bile acid deconjugation and transformation into secondary bile acids [11], thereby affecting host metabolic signalling.

While previous studies have largely focused on changes in gut microbiota composition following fruit or fibre intake, fewer have examined the resulting metabolic outputs of these interactions. Faecal metabolomics, or stool metabolite profiling, captures the spectrum of low-molecular-weight compounds generated through microbial fermentation and host–microbe co-metabolism activities [26]. Proton nuclear magnetic resonance (^1^H NMR) spectroscopy provides a robust platform for quantifying and annotating untargeted small metabolites originating from dietary inputs and microbial transformations [27]. Despite considerable research on apples, few studies have examined the immediate and food-realistic intake of apples on the human faecal metabolomic profile. Prior studies have focused predominantly on long-term consumption, systemic biomarkers and in vitro models [28,29,30].

The present study was designed as a controlled exploratory dietary intervention to investigate the effects of short-term consumption of a standardised, food-typical quantity of apples cv. Gala on the human faecal metabolomic profile. On the basis that faeces can reflect luminal biochemical activity, the study aimed to determine whether intake of a defined apple cultivar is associated with measurable changes in gut metabolic processes. We hypothesised that short-term apple consumption may modulate microbial fermentation patterns, as reflected by alterations in faecal metabolites associated with saccharolytic and proteolytic pathways.

## 2. Materials and Methods

### 2.1. Subjects

Fifteen subjects (*n* = 15) were selected for this pilot interventional study, comprising six females and nine males aged 18–30 years (mean ± SD: 23.4 ± 3.76 years). Thirteen participants had a normal weight (BMI 18–25), while the remaining two were overweight (BMI 25–30). The overall mean BMI of the study population was 23.56 ± 2.55, reflecting a predominantly normal-weight cohort with limited representation of overweight individuals. Detailed subject-level demographic and anthropometric data are provided in Supplementary Table S1. Eight participants had active jobs, while the rest had sedentary jobs. All the participants were physically active, engaging in some form of sport on an average of 4.13 ± 2.85 h/week. None of the participants were on a specific diet or medications and they were not selected based on their habitual apple consumption. The study followed the principles of the Declaration of Helsinki and received approval from the Ethics Committee for Multicentric Evaluation, Regional Hospital Liberec, a.s., Czech Republic (Ref. No. EK/81/2023, approval date: 25 October 2023). All personal data was handled in compliance with the EU General Data Protection Regulation (GDPR), and all participants signed a consent form. This study was registered retrospectively at ClinicalTrials.gov (U.S. National Library of Medicine, Bethesda, MD, USA; Identifier NCT07499960; registered on 24 March 2026).

### 2.2. Apple Intervention

**Figure 1:**
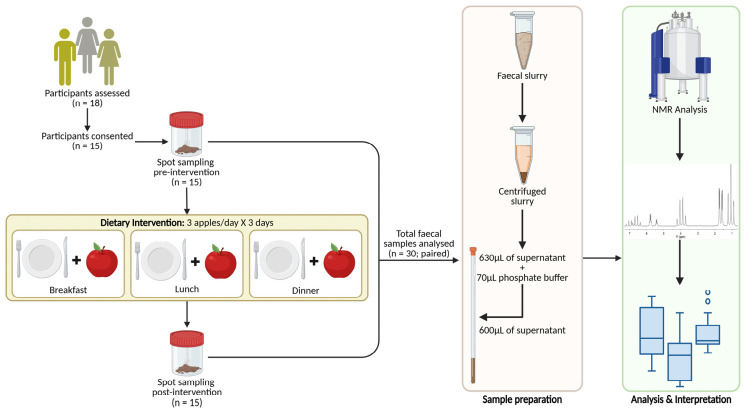
Study design and workflow of the short-term Gala apple intervention. Participants consumed three Gala apples daily (one per main meal) for three consecutive days while maintaining their habitual diet. Faecal samples were collected before (baseline) and after the intervention and analysed using ^1^H NMR-base metabolomics.

Apples of the Gala cultivar were obtained from a local farm and distributed to all participants prior to the start of the intervention. No wash-out period was implemented before the interventional period. For three consecutive days, participants incorporated three apples daily into their habitual diet. One Gala apple was consumed with each main meal (breakfast, lunch, and dinner), without modification of other dietary components (Figure 1).

The selected intake (3 apples/day) was designed to provide a standardised and sufficiently elevated dietary exposure within a short timeframe, enabling the detection of acute metabolomic responses. Based on typical values, this corresponds to an estimated dietary fibre intake of approximately 9−11 g/day, representing a substantial proportion (35−45%) of the Adequate Intake for fibre (25 g/day) established by the EFSA [17,31]. While this intake may exceed habitual consumption levels for some individuals, it remains within range of short-term increased fruit-intake and was chosen to ensure a nutritionally meaningful and measurable exposure.

The 3-day intervention was designed to cover the typical gastrointestinal transit time (approximately 24–72 h), thereby increasing the likelihood that metabolites derived from Gala apple consumption would be present in the post-intervention faecal samples [32]. Accordingly, the observed metabolomic changes likely reflect recent dietary exposure to apples rather than longer-term adaptation of the gut microbiota.

### 2.3. Sample Collection and Preparation

Each participant self-collected a spot faecal sample on the day prior to the 3-day intervention and another spot sample on the day following completion of the intervention. Participants received a stool collection kit containing a plastic container with a screw-cap lid and an integrated sampling spoon, disposable collection paper, and detailed written instructions for proper use. After collection, the faecal samples were immediately frozen at −18 °C by the participants and transported to the laboratory under cold-chain conditions. Upon arrival at the analysis site, samples were stored at −80 °C until analysis. In total, 30 samples were processed for metabolomic assessment, and faecal metabolite concentrations were compared between pre- and post-intervention time points.

For the ^1^H NMR-based metabolomics analysis, 200–250 mg of each collected specimen was weighed after thawing and transferred into new microtubes. Ultrapure water (1 mL) was added to each sample to form a slurry, which was vortexed for at least 2 min to achieve complete homogenisation. Samples were then centrifuged at 24,400× *g* for 15 min at 4 °C to sediment particulate matter. From the resulting supernatant, 630 µL was combined with 70 µL of phosphate buffer in D_2_O (1.5 M K_2_HPO_4_/1.5 M NaH_2_PO_4_, 5 mM 3-(trimethylsilyl)-2,2,3,3-tetradeuteropropionic acid (TSP), 0.2% NaN_3_, pH 7.4) in fresh microtubes. The mixtures were vortexed for 1 min and centrifuged again at 24,400× *g* for 10 min. Finally, 600 µL of the clarified supernatant was transferred into 5 mm NMR tubes (high-throughput, 7-inch, Norell^®^, Morganton, NC, USA) for spectral acquisition. The phosphate buffer pH 7.4 was used to ensure consistent spectral conditions and adjust for small pH differences between samples. Faecal pH was not measured. All chemicals were of analytical grade and purchased from Sigma-Aldrich (St. Louis, MA, USA). To minimise technical variability, all samples were randomised prior to processing and prepared within a single sample preparation session. Subsequent ^1^H NMR analyses were performed under identical instrumental conditions.

### 2.4. Sample Analysis and Data Acquisition

^1^H NMR spectra were acquired at 298 K on a 500 MHz Bruker Avance III spectrometer (Bruker BioSpin GmbH, Rheinstetten, Germany) equipped with a BBFO SmartProbe™ with Z-axis gradients and a 60-position autosampler (SampleCase™, Bruker). Water suppression was achieved using One-Dimensional Nuclear Overhauser Effect Spectroscopy Nuclear Magnetic Resonance pulse sequence with water presaturation (Bruker noesypr1d pulse sequence) with t1 set to 4 µs and dmix to 0.1 s. Spectra were collected with 16 dummy scans, 128 scans, a spectral width of 16.02 ppm from 64 k data points, a 4.09 s acquisition time, and a 1 s relaxation delay. Automated probe tuning, 3D shimming, and 90° pulse calibration were carried out before each acquisition, and receiver gain was kept constant across all measurements. Spectra were evaluated according to the linewidth of the TSP reference signal (linewidths < 1 Hz).

Initial data processing was performed in MNova (version 14.1.0, Mestrelab Research, Santiago de Compostela, Spain). Free induction decays (FIDs) were zero-filled to 128 k data points and Fourier-transformed using an exponential window function with a 0.5 Hz line-broadening factor. Manual phase correction and baseline adjustment (Whitaker smoother) were applied in MNova. The processed spectra were exported in .jcamp format and imported into Chenomx NMR Suite (version 9.01, Chenomx Inc., Edmonton, AB, Canada). Metabolite profiling was performed in Chenomx Profiler, where a total of 67 metabolites were annotated and quantified by spectral fitting relative to the known concentration of the internal TSP reference [33,34]. Zero values in metabolite concentrations, representing below the limit of detection, were imputed prior to statistical analysis. For each metabolite, zero values were replaced with one-third of the minimum non-zero concentration observed for that metabolite across all samples. Metabolite concentrations were exported from Chenomx and converted to µg/g in wet stool samples by accounting for sample weight, the respective dilution factors, and extraction volumes.

As an additional layer of investigation, the following parameters were considered: the sum of SCFAs (acetate, butyrate, propionate), partial sums (acetate and propionate, acetate and butyrate), the sum of branched-chain fatty acids (BCFAs; 2-hydroxy-3-methylvalerate, isovalerate, isobutyrate, and 2-methylbutyrate), the combined concentration of valine, leucine, and isoleucine, and the ratio between total SCFAs and BCFAs. These composite variables were included to account for the functional shifts in saccharolytic and proteolytic fermentation pathways [35,36,37,38].

### 2.5. Statistical Analysis and Data Interpretation

All statistical analyses were conducted in R (version 4.4.2; R Foundation for Statistical Computing, Vienna, Austria) [39]. All metabolites were naturally log-transformed prior to analysis. Principal component analysis (PCA) was performed on the transformed metabolite data using the prcomp function, following mean-centering, and scaling to unit variance. Variable contributions to the first five principal components were examined, and sample score–loading plots were generated with the factoextra package (version 1.0.7) [40].

To account for repeated measures and inter-individual variability, linear mixed models (LMMs) were fitted using the lmerTest package (version 3.1-3) [41], with intervention specified as a fixed effect and subject as a random effect. Resulting *p*-values (*p*) were adjusted for multiple testing (q) using the Benjamini–Hochberg procedure, and values < 0.05 were considered nominally significant. Fold changes (FC) were estimated via antilog of the estimated change in the dependent variable (β). Data visualisations were produced in ggplot2 (version 3.5.0) [42] with customised themes to ensure clarity and consistency.

## 3. Results

### 3.1. Identification and Annotation of the Faecal Metabolomic Profile

A comprehensive ^1^H NMR-based analysis of faecal samples collected before and after the apple-intake intervention resulted in the identification and annotation of 67 metabolites including amino acids, organic acids, SCFAs, and other diet- and host-derived metabolites (Supplementary Materials: Table S2, Figures S1–S3).

**Figure 2:**
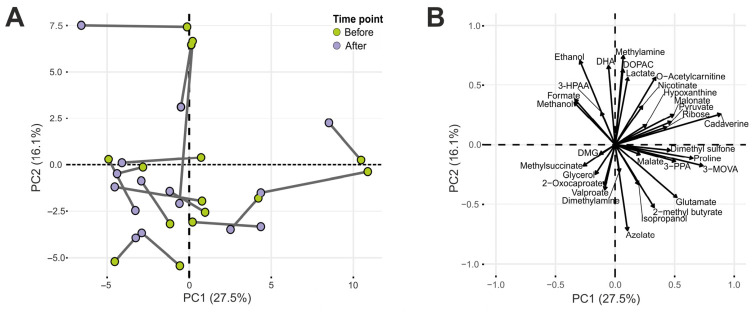
Principal component analysis (PCA) of metabolites detected via ^1^H NMR spectroscopy in faecal sample before and after apple-intake intervention. (**A**) Score plot. Samples from the same subjects are connected with a line. (**B**) Loading plot. For clarity, the biplot displays only the 30 variables with the highest loadings, while the PCA was performed using all variables. Abbreviations used: 3-HPAA: 3-hydroxyphenylacetate; DMG: *N*,*N*-dimethylglycine; 3-MOVA: 3-methyl-2-oxovalerate; DOPAC: 3,4-dihydroxyphenylacetate; 3-PPA: 3-phenylpropionate; DHA: 1,3-dihydroxyacetone.

Global metabolic variation was assessed using principal component analysis (PCA), which revealed no distinct clustering between pre- and post-intervention. The first two principal components explained 43.6% of total variance (Figure 2), indicating that inter-individual variability exceeded intervention-driven changes. Accordingly, subsequent analyses focused on univariate and mixed-model approaches to detect metabolite-specific changes.

### 3.2. Effect of Gala Apple Consumption on Metabolic Profile

**Figure 3:**
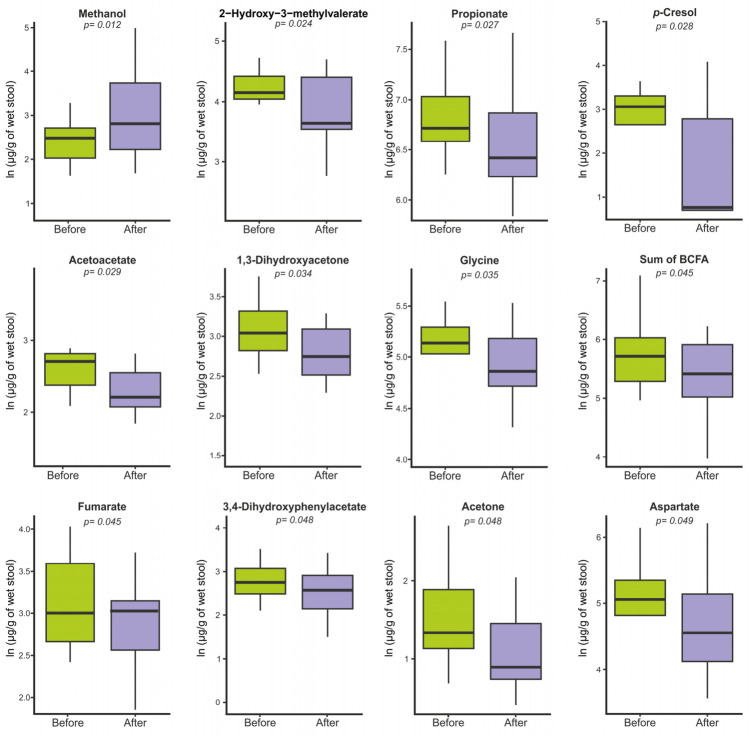
Faecal metabolite concentrations before and after the apple-intake intervention. Each box plot represents the distribution of natural log-transformed metabolite concentrations (µg/g of wet stool). *p*-values were derived from linear mixed-effects model (LMM) analysis. Abbreviations used: BCFA—Branched-chain fatty acid.

Differential analysis identified methanol as the only metabolite showing a nominal increase following the intervention (β = +0.72, FC ≈ 2.06, *p* = 0.012), although this effect did not remain statistically significant after Benjamini–Hochberg correction for multiple testing (q = 0.27; Figure 3). All other metabolites exhibited decreasing trends with nominal significance, but none retained significance after correction (all q = 0.27) (Figure 3). Given the exploratory nature of this pilot study, metabolites showing consistent directionality with nominal significance (*p* < 0.05) are reported, even when not retained after correction for multiple testing.

Specifically, nominal reductions were observed in amino acids including glycine (β = −0.19, FC = 0.83, *p* = 0.035, q = 0.27), aspartate (β = −0.25, FC = 0.78, *p* = 0.049, q = 0.27), and tryptophan (β = −0.39, FC = 0.68, *p* = 0.050, q = 0.27), as well as in BCFAs, namely 2-hydroxy-3-methylvalerate (β = −0.42, FC = 0.66, *p* = 0.024, q = 0.27) and total BCFAs (β = −0.30, FC = 0.74, *p* = 0.045, q = 0.27). Decreases were also detected for the SCFA propionate (β = −0.22, FC = 0.80, *p* = 0.027, q = 0.27) and for energy-related intermediates such as acetoacetate (β = −0.32, FC = 0.72, *p* = 0.029, q = 0.27) and 1,3-dihydroxyacetone (β = −0.31, FC = 0.73, *p* = 0.034, q = 0.27). Finally, aromatic microbial metabolites including *p*-cresol (β = −1.04, FC = 0.35, *p* = 0.028, q = 0.27) and 3,4-dihydroxyphenylacetate (β = −0.48, FC = 0.62, *p* = 0.048, q = 0.27) were also reduced. However, none of these changes remained statistically significant after Benjamini–Hochberg correction for multiple testing.

## 4. Discussion

In this pilot intervention study, three days of increased Gala apple intake was associated with a ≈ 2.05-fold rise in faecal methanol, along with decreases in selected amino acids, BCFAs, SCFAs, energy-related intermediates, and aromatic microbial metabolites. PCA showed that inter-individuality outweighed global intervention effects, the metabolite-specific patterns point towards a subtle modification of gut microbial metabolism in response to short-term fruit-derived fibre intake [2,3,8,9,10,11,12,21,22,25,43]. However, none of the observed metabolite changes remained statistically significant after correction of multiple testing (q ≈ 0.27). Furthermore, due to the absence of a control group, the causal relationships between Gala apple consumption and the observed metabolomic changes cannot be established. Hence, these findings therefore be interpreted as exploratory, requiring validation in larger controlled studies.

The most prominent observation was the ≈ 2-fold increase in faecal methanol following apple consumption. This is consistent with the known metabolic fate of pectin, the major soluble fibre in apples, which largely escapes digestion in the upper gastrointestinal tract and reaches the colon where it is depolymerised and demethylated by pectin-degrading bacteria [18,19,20,44,45,46,47]. Bacterial pectin methylesterases (PMEs) cleave methyl-esterified galacturonic acid residues, releasing methanol as a stoichiometric by-product of demethylation [44,45,46,48,49]. Methanol formation may also be affected by endogenous PMEs present in many fruits, which catalyse pectin de-esterification before and during digestion and may therefore contribute alongside host and microbial enzymatic processes [48]. In addition to this, several members of the Bacillota, Bacteroidota, and Actinomycetota phyla harbour pectinolytic capacities and form key hubs in colonic carbohydrate degradation [46,47]. In habitual diet, methanol may also originate directly from dietary sources, as it is naturally present in fruits and vegetables, beverages and endogenous metabolic processes in the host [50,51]. These observations align with previous work on fruit- and fibre-based interventions. Human studies have further demonstrated that consumption of fresh fruits and vegetables can acutely increase blood methanol concentrations by 20–30% [50,52]. Therefore, diet derived methanol is increasingly viewed as a normal component of plant-rich diet rather than and toxicant per se [50,51,52,53]. While this observation suggests that methanol could serve as a functional indicator of pectin fermentation, its specificity and applicability as a biomarker of fruit fibre intake, although this may require validation in larger and more diverse cohorts.

Beyond methanol, apple consumption was also associated with consistent reductions in several metabolites linked to proteolytic fermentation and microbial degradation of aromatic substrates. Concentrations of glycine, tryptophan, and aspartate, as well as 2-hydroxy-3-methylvalerate and total BCFAs, tended to decrease following intervention. In parallel, we observed lower faecal levels of *p*-cresol and 3,4-dihydroxyphenylacetate, both of which are the products of microbial transformation of aromatic amino acids and (poly)phenols. Although none of these changes remain statistically significant after correction for multiple testing and should therefore be interpreted with caution.

The reduction in amino acids and amino acid degradation products is noteworthy and may reflect a shift in the balance between proteolytic and saccharolytic fermentation. In general, higher protein intake is associated with increased luminal amino acids and proteolytic metabolites, whereas higher fibre intake tends to promote saccharolytic fermentation, SCFA production, and related changes in microbial composition [54,55]. This may underline the pattern observed here. Although no increase in faecal SCFAs was detected, this does not exclude enhanced saccharolytic fermentation, since SCFAs are extensively absorbed and metabolised in the colon and faecal concentrations may not accurately represent intestinal production as seen in other studies [56,57]. Thus, the observed decline in amino acids and amino acid degradation products may still be compatible with a shift toward saccharolytic metabolism.

An alternative explanation is that increased apple intake displaced protein-rich foods from the diet, with participants potentially replacing foods such as milk or meat products with fibre-rich apples due to greater satiety. The present study design does not allow these two explanations to be disentangled. Nevertheless, similar observations have been reported in comparable dietary intervention studies, especially those involving increased fruit consumption.

Previous studies using mango, banana, and kiwifruit have reported consistent shifts with enhanced fibre utilisation and reduced proteolytic outputs, often accompanied by modest changes in faecal SCFA, highlighting improvements in fermentation balance [25,37,38]. Likewise, inclusion of apple pectin or low levels of mango peel in feed has been shown to modulate in vitro fermentability, reduce *p*-cresol formation, reduce amino acids, and alter SCFA and BCFA patterns in directions generally interpreted as beneficial for colonic health [47,58,59].

Contrary to our initial hypothesis that increased fermentable substrate availability might elevate faecal SCFA levels, we did not observe an increase in total SCFAs. Instead, propionate and total SCFAs exhibited a modest (≈10%) decrease, along with a subtle decline in energy-related intermediates such as acetoacetate and 1,3-dihydroxyactone. This apparent discrepancy does not necessarily contradict enhanced saccharolytic activity. Faecal SCFA concentrations represent the net balance of production, luminal utilisation, absorption by colonocytes, and transit time, rather than production alone [21,22,25]. Increased proximal SCFA production can be accompanied by greater epithelial uptake and oxidation, resulting in lower or unchanged faecal SCFA levels despite higher metabolic flux. Several fruit and fibre interventions have reported either stable SCFAs or modest shifts in their distribution, alongside a clear reduction in proteolytic metabolites and improvements in host outcomes [3,21,43,60].

This study focused on Gala, a widely consumed commercial apple cultivar. Although some compositional variation exists among apple cultivars, including differences in dietary fibre, pectin, polyphenol composition, and organic acids [61,62], which are further influenced by growing conditions and post-harvest factors, Gala is not compositionally extreme. Rather, it falls within the typical range of commercially available cultivars, without pronounced deviations in fibre or phytochemical content compared with other commonly consumed apples [31,63].

This study has several limitations. The faecal metabolome is influenced by multiple factors beyond diet, including age, sex, body composition, habitual dietary patterns, and lifestyle choices. Although the within-subject design partially accounts for inter-individual variability, these factors cannot be fully controlled. In addition, the relatively small sample size, absence of control group, and lack of cross-over design limit the generalisability of the findings. The absence of wash-out period may contribute to baseline variability; however, it reflects the intentional design of the study where participants maintained habitual diets. Although, the 3-day intervention may initially be perceived as a limitation, this duration was deliberately selected and should be considered a strength of the study design. Specifically, the intervention period was chosen to align with gastrointestinal transit time (approximately 24–72 h), enabling the detection of metabolites associated with apple consumption in faecal samples. The aim of the study was not to capture longer-term adaptations in gut microbiota composition, but rather to characterise the short-term metabolic response to apple intake as reflected in the faecal metabolome. Finally, while NMR-based metabolomics provide robust and quantitative profiling of major metabolites, broader metabolite coverage using complementary analytical platforms such as LC-MS could further enhance sensitivity and resolution. Future studies involving larger and more diverse cohorts, longer intervention periods, as well as mechanistic and integrated multi-omics approaches will be important to validate and extend these findings.

## 5. Conclusions

In this pilot study, short-term apple consumption was associated with nominal, metabolite-specific changes in the faecal metabolome, with no clear global shifts. An increase in faecal methanol, alongside modest decreases in selected amino acids, BCFAs, aromatic microbial metabolites, were observed. However, these changes did not remain significant after correction for multiple testing.

These patterns may reflect a subtle modulation of gut microbial metabolism in response to short-term apple-derived fibre intake. However, given the exploratory design, small sample size, and high inter-individual variability, causal relationships cannot be established. Overall, this study highlights the potential of faecal metabolomics to capture short-term diet-related metabolic responses, while emphasising the need for larger, controlled studies incorporating complementary approaches to confirm and extend these observations.

## Data Availability

The data are not publicly available due to ethical and privacy restrictions associated with human participant data and institutional regulations. The raw data supporting the conclusions of this article will be made available by the authors on request.

## Supplementary Materials

The following supporting information can be downloaded at: https://www.mdpi.com/article/10.3390/nu18091312/s1. Table S1: Baseline characteristics of study subjects. Table S2: List of metabolites detected in faecal samples by ^1^H NMR spectroscopy and their assigned resonance chemical shifts (ppm). Figure S1: Representative aromatic region of the ^1^H NMR spectrum of a fecal sample, showing the annotated metabolites included in the study. Figure S2: Representative carbohydrates region of the ^1^H NMR spectrum of a fecal sample, showing the annotated metabolites included in the study. Figure S3: Representative aliphatic region of the ^1^H NMR spectrum of a fecal sample, showing the annotated metabolites included in the study.

## Abbreviations

The following abbreviations are used in this manuscript:

LMM	Linear mixed-effects model
PCA	Principal Component Analysis
SCFA	Short-chain fatty acid
BCFA	Branched-chain fatty acid
3-HPAA	3-hydroxyphenylacetate
DMG	*N*,*N*-dimethylglycine
3MOVA	3-methyl-2-oxovalerate
DOPAC	3,4-dihydroxyphenylacetate
3-PPA	3-phenylpropionate
DHA	1,3-dihydroxyacetone
