# Supplementary material for: Impact of Short-Term Gala Apple Intake on the Human Faecal Metabolome Assessed by 1H NMR Spectroscopy
Source: Nutrients. 2026 Apr 22;18(9):1312. doi: 10.3390/nu18091312 (PMC13164819; doi:10.3390/nu18091312)
Supplement: Supplementary file 1 [file nutrients-18-01312-s001.zip › nutrients-4237101-supplementary.pdf]

Supplementary material

Impact of short-term apple intake on the human faecal metabolome assessed by ^{1}H NMR spectroscopy

Chandrama Roy Chowdhury[1], Anna Mascellani Bergo[1], Eliška Jeníčková[1], Šárka Knížková[1] and Jaroslav Havlík[1]*

[1] Department of Food Science, Czech University of Life Sciences Prague
* Correspondence: havlik@af.czu.cz; Tel.: +420-224382965

Table S1: Baseline characteristics of study subjects

Subject Code	Gender	Age (years)	Height (cm)	Weight (kg)	BMI
1001	Male	24	192	100	27.13
1002	Male	23	172	65	21.97
1003	Male	30	186	65	18.79
1004	Male	30	174	85	28.08
1005	Female	18	167	65	23.31
1006	Female	24	166	54	19.60
1007	Male	24	181	82	25.03
1008	Male	23	182	80	24.15
1009	Male	24	179	80	24.97
1010	Male	26	185	81	23.67
1011	Female	22	177	68	21.71
1012	Male	21	187	75	21.45
1013	Female	18	170	74	25.61
1014	Female	18	152	55	23.81
1015	Female	26	168	68	24.09

Table S2: List of metabolites detected in faecal samples by ^{1}H NMR spectroscopy and their assigned resonance chemical shifts (ppm).

Metabolite	Characteristic resonance δ (ppm, multiplicity)
1,3-Dihydroxyacetone	4.41 (m), 3.57 (m)
2-Hydroxy-3-methylvalerate	0.93 (d), 0.85 (d)
2-Hydroxyisovalerate	0.95 (d), 0.82 (d)
2-Methylbutyrate	0.90 (t)
2-Oxocaproate	0.88 (t)
2-Oxoisocaproate	0.93 (d)
3-(3-Hydroxyphenyl)propionate	7.23–6.76 (aromatic m), 2.82 (t), 2.46 (t)
3,4-Dihydroxyphenylacetate	6.86–6.71 (aromatic m), 3.38 (s)
3-Hydroxyphenylacetate	7.26–6.78 (aromatic m), 3.47 (s)
3-Methyl-2-oxovalerate	0.88 (d)
3-Phenylpropionate	7.36–7.25 (aromatic m), 2.88 (t), 2.48 (t)
4-Hydroxyphenylacetate	7.15 (d), 6.85 (d), 3.42 (s)
Acetate	1.91 (s)
Acetoacetate	3.44 (s), 2.27 (s)

Acetone	2.22 (s)
Alanine	3.77 (q), 1.47 (d)
Arabinose	5.24 (d), sugar region multiplets
Aspartate	2.80 (dd), 2.67 (dd)
Azelate	2.17 (t), 1.29 (m)
Butyrate	2.15 (t), 0.88 (t)
Cadaverine	3.02 (t), 1.72 (m)
Dimethyl sulfone	3.14 (s)
Dimethylamine	2.71 (s)
Ethanol	3.65 (q), 1.17 (t)
Formate	8.44 (s)
Fucose	1.24 (d), sugar multiplets
Fumarate	6.51 (s)
Galactose	5.26 (d), sugar multiplets
Glucose	5.23 (d), sugar multiplets
Glutamate	2.36 (m), 2.12 (m)
Glycerol	3.78–3.55 (m)
Glycine	3.55 (s)
Hypoxanthine	8.20 (s), 8.18 (s)
Isobutyrate	2.38 (m), 1.05 (d)
Isoleucine	1.00 (d), 0.93 (t)
Isopropanol	4.01 (sept), 1.16 (d)
Isovalerate	0.90 (d)
Lactate	4.11 (q), 1.32 (d)
Leucine	0.95 (d), 0.94 (d)
Malate	4.29 (dd), 2.66 (dd)
Malonate	3.12 (s)
Methanol	3.35 (s)
Methionine	2.63 (t), 2.15 (m)
Methylamine	2.59 (s)
Methylsuccinate	2.62 (m), 1.07 (d)
N,N-Dimethylglycine	3.71 (s), 2.91 (s)
N-Acetyltyrosine	7.72 (d), 1.92 (s)
Nicotinate	8.93 (s), 8.60 (s)
O-Acetylcarnitine	3.20 (s), 2.13 (s)
p-Cresol	7.13 (d), 6.82 (d), 2.25 (s)
Phenylacetate	7.37–7.29 (aromatic m), 3.53 (s)
Phenylalanine	7.42–7.32 (aromatic m), 3.98 (m)
Proline	4.12 (m), 2.02 (m)
Propionate	2.17 (q), 1.04 (t)
Propylene glycol	3.87 (m), 1.13 (d)
Pyruvate	2.36 (s)
Ribose	5.38 (d), sugar multiplets
Succinate	2.39 (s)
Threonine	4.24 (d), 1.32 (d)

Trimethylamine-N-oxide	3.25 (s)
Tryptophan	10.18 (s), aromatic multiplets
Tyrosine	7.18 (d), 6.89 (d)
Uracil	7.53 (d), 5.79 (d)
Valerate	2.17 (t), 0.88 (t)
Valine	1.03 (d), 0.98 (d)
Valproate	0.86 (t)
Xanthine	7.90 (s)

Note: Chemical shift values are reported in parts per million (ppm) and referenced to the internal standard trimethylsilylpropionic acid (TSP, δ = 0.00 ppm). Multiplicity of resonances is indicated in parentheses where applicable.

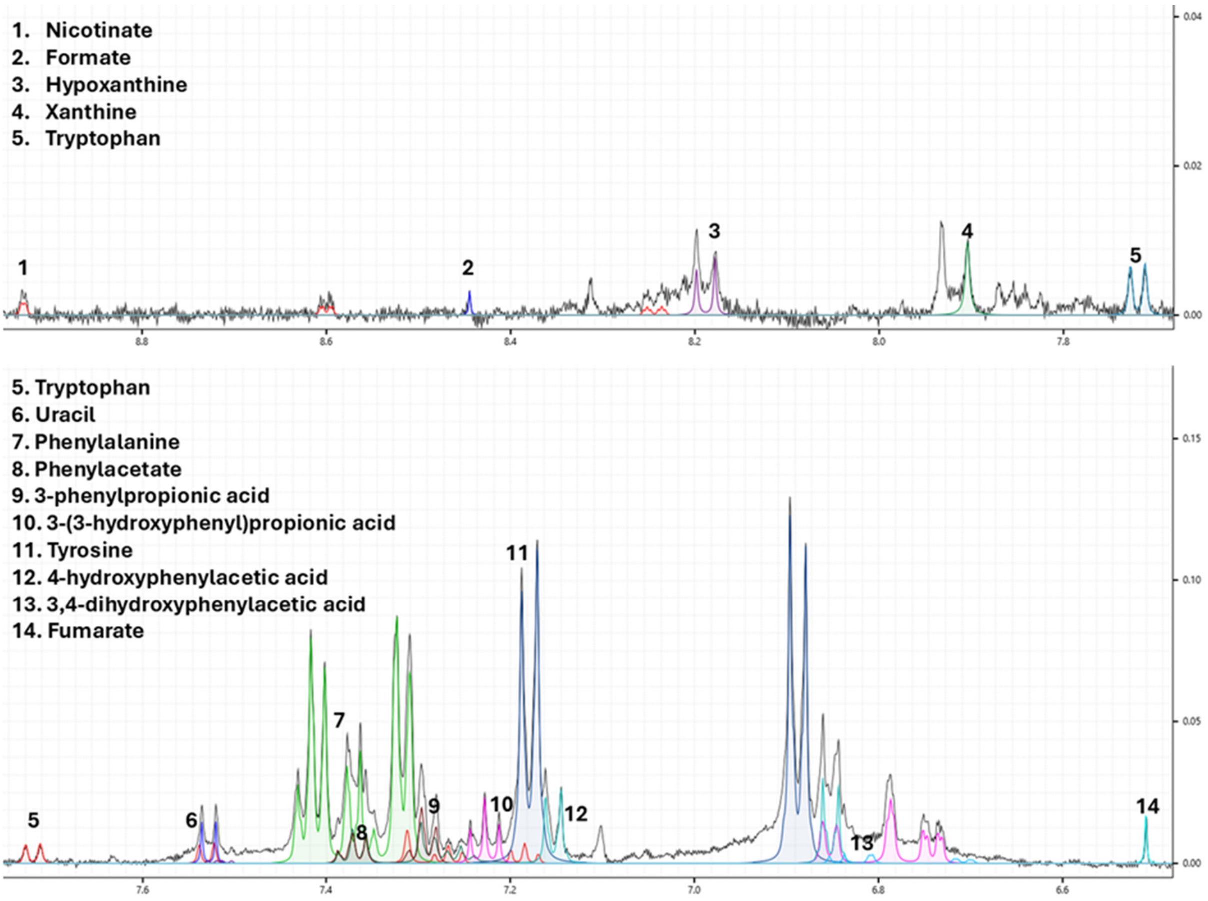

Figure S1: Representative aromatic region of the ¹H NMR spectrum of a fecal sample, showing the annotated metabolites included in the study. For clarity, only selected representative metabolites are annotated in the spectrum. A complete list of identified compounds is provided in Table S2.

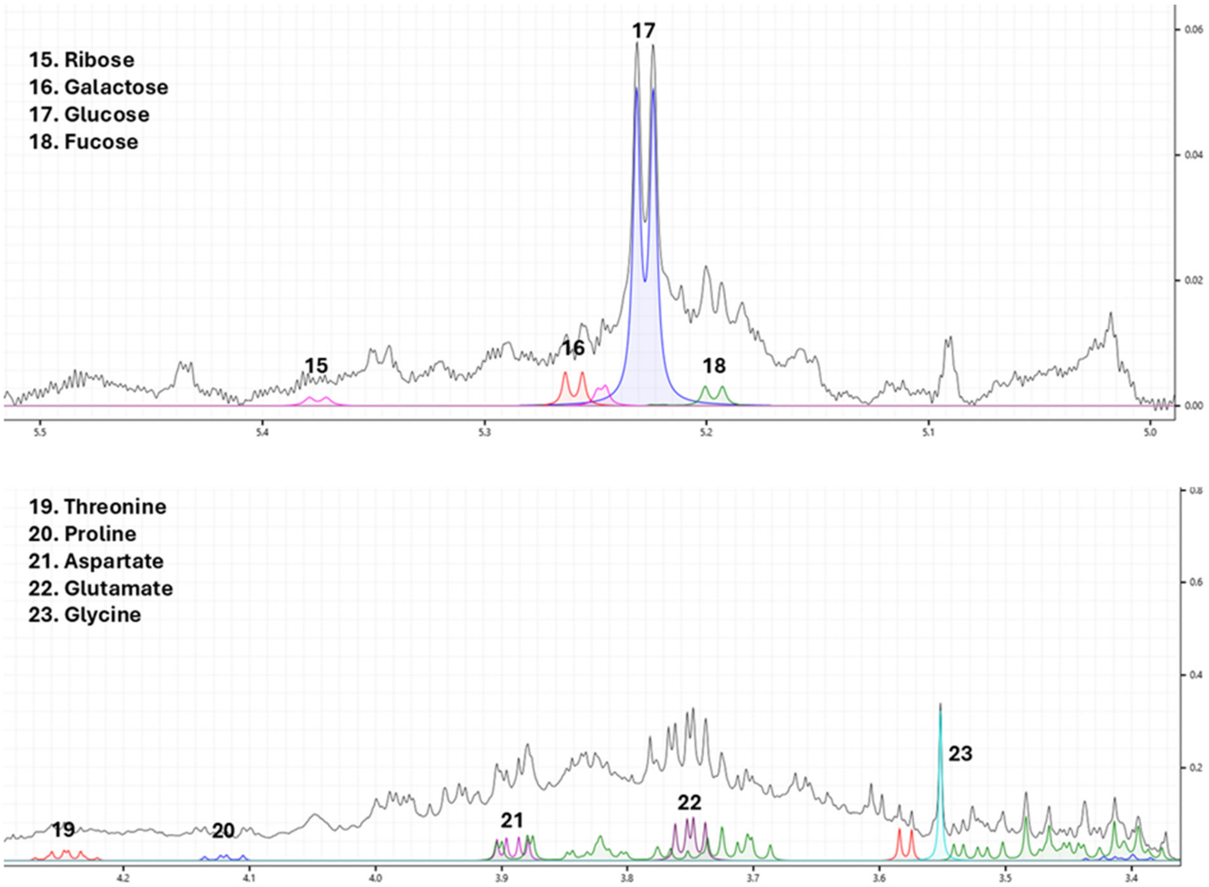

Figure S2: Representative carbohydrates region of the ^{1}H NMR spectrum of a fecal sample, showing the annotated metabolites included in the study. For clarity, only selected representative metabolites are annotated in the spectrum. A complete list of identified compounds is provided in Table S2.

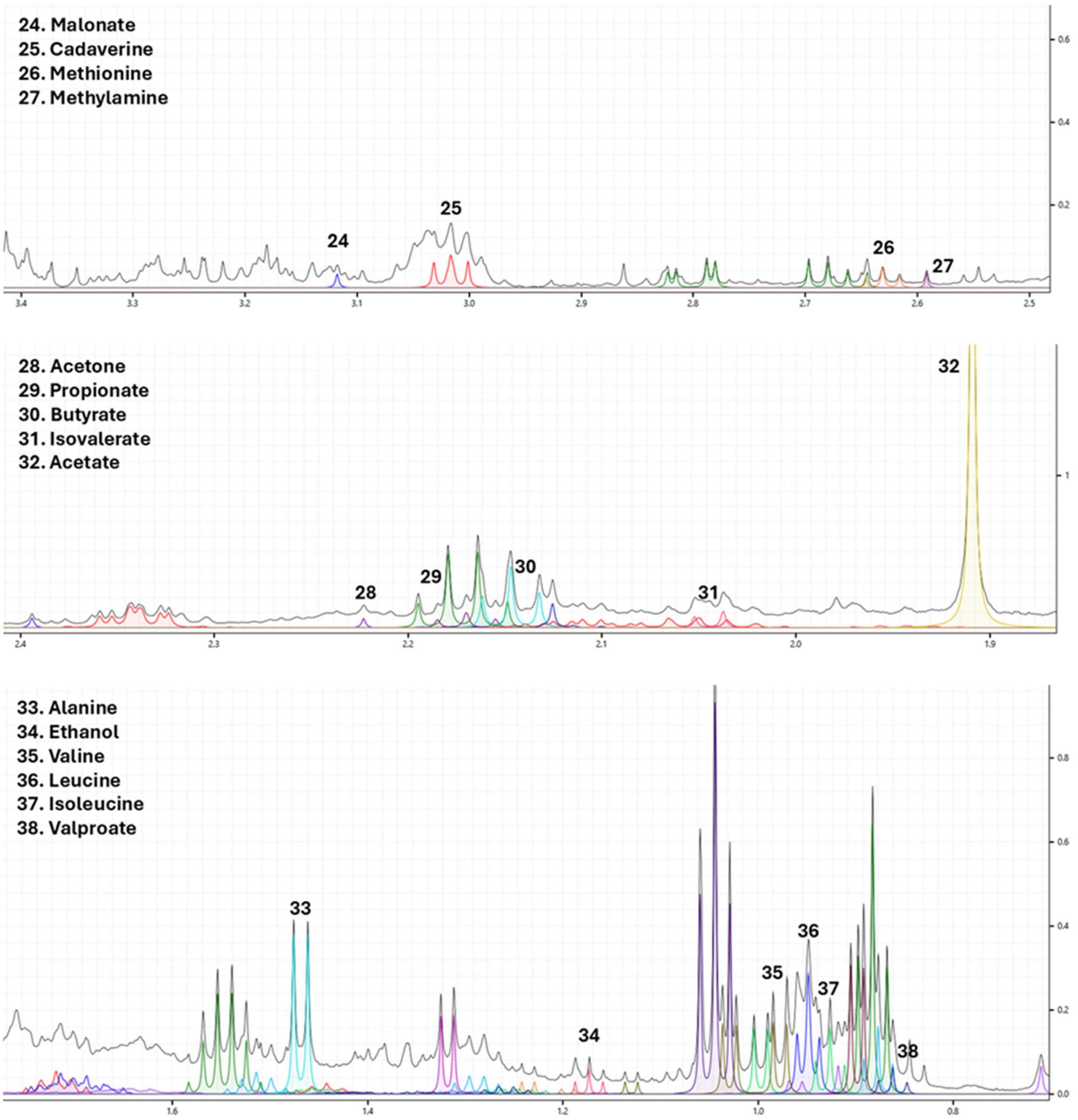

Figure S3: Representative aliphatic region of the ^{1}H NMR spectrum of a fecal sample, showing the annotated metabolites included in the study. For clarity, only selected representative metabolites are annotated in the spectrum. A complete list of identified compounds is provided in Table S2.
